# A Fatal Combination: A Thymidylate Synthase Inhibitor with DNA Damaging Activity

**DOI:** 10.1371/journal.pone.0117459

**Published:** 2015-02-11

**Authors:** Anna Ligasová, Dmytro Strunin, David Friedecký, Tomáš Adam, Karel Koberna

**Affiliations:** Institute of Molecular and Translational Medicine, Faculty of Medicine, Palacký University, Olomouc, 779 00, Czech Republic; Institute of Enzymology of the Hungarian Academy of Science, HUNGARY

## Abstract

2′-deoxy-5-ethynyluridine (EdU) has been previously shown to be a cell poison whose toxicity depends on the particular cell line. The reason is not known. Our data indicates that different efficiency of EdU incorporation plays an important role. The EdU-mediated toxicity was elevated by the inhibition of 2′-deoxythymidine 5′-monophosphate synthesis. EdU incorporation resulted in abnormalities of the cell cycle including the slowdown of the S phase and a decrease in DNA synthesis. The slowdown but not the cessation of the first cell division after EdU administration was observed in all of the tested cell lines. In HeLa cells, a 10 μM EdU concentration led to the cell death in the 100% of cells probably due to the activation of an intra S phase checkpoint in the subsequent S phase. Our data also indicates that this EdU concentration induces interstrand DNA crosslinks in HeLa cells. We suppose that these crosslinks are the primary DNA damage resulting in cell death. According to our results, the EdU-mediated toxicity is further increased by the inhibition of thymidylate synthase by EdU itself at its higher concentrations.

## Introduction

The use of 2′-deoxy-5-ethynyluridine (EdU) as an anti-viral substance was already studied in the nineteen seventies [1,2]. Although this analogue of 2′-deoxyuridine evinced an anti-HSV-1 and HSV-2 (Herpes simplex virus) effect and also an impact against the vaccinia virus, the effective concentration also inhibited the growth and metabolism of non-infectious cells [1]. Similar results were also obtained in 2007 in the case of cytomegalovirus [3]. In this case, the effective concentration required to reduce the cell growth of human embryonic lung cells by 50% was 2.5 μM while the inhibitory concentration required to reduce virus-plaque formation in these cells by 50% was 0.85–1.2 μM [3]. It was simultaneously shown that the inhibitory effect on the proliferation of FM3A/O and FM3A*tk-*/HSV-1*tk+* was higher on cells with viral thymidine kinase [3]. EdU was also successfully tested as a possible inhibitor of the cell growth of human breast cancer cells (MCF-7 and MDA-MP-231) with the IC50 of 0.4 μM for MCF-7 cells and 4.4 μM for MDA-MB-231 cells [4]. The mechanism of the inhibition, however, remained unknown, although some of the data indicated that EdU can act as an inhibitor of thymidylate synthase [5].

The interest in EdU was greatly revived in 2008 when this nucleoside analogue was used as a marker of cellular replicational activity [6]. Due to its simple and fast visualization, EdU immediately became a very strong competitor of the most frequently used marker to date nucleoside—5-bromo-2′-deoxyuridine (BrdU). In contrast to BrdU detection based on the use of specific antibodies, the reaction between the azido group of the tag molecule and the ethynyl group of EdU is employed in EdU detection [6]. This reaction is catalysed by the monovalent copper ions and is performed without any additional steps. In contrast, BrdU visualisation requires special steps leading to its revelation in the DNA structure [7–11]. Due to the renewed interest in EdU and the high number of cell lines used in various studies, new findings about the impact of EdU on cell metabolism were obtained. The data of Ross and colleagues [12] indicated that EdU incorporation can lead to DNA breaks followed by cell death. Simultaneously, they also showed that EdU supresses in vitro population expansion and in vivo tumour progression in human glioblastoma cells [12]. On the bases of immunolocalisation studies of the proteins γH2AX and p53BP1 it was suggested that EdU induces double-stranded DNA breaks as well [13]. Although it is evident that EdU toxicity is highly dependent on the cell line used [3,4,13–15], the reason for the different effect of EdU in various cell lines remained unknown.

In the study presented, we have focused on the possibility that the different cytotoxic effect of EdU could be related to the different rate of EdU incorporation in DNA. We also studied (i) the changes in the rate of DNA replication and cell cycle progression, (ii) the possibility that EdU can generate interstrand crosslinks and (iii) the role of the metabolism of 2′-deoxythymidine (dT) in EdU-mediated toxicity. Overall, our data indicated that

- EdU toxicity positively correlates with the efficiency of its incorporation and this efficiency is different in different cell lines.
- The incorporation of EdU is dependent on the intracellular concentrations of dT and 2′-deoxythymidine 5′-monophosphate (dTMP).
- EdU incorporation in DNA leads to the deceleration and deformation of the cell cycle including the slowdown of the S phase accompanied by a decrease in the DNA synthetic activity.
- Although the in vivo inhibitory effect of EdU on the activity of thymidylate synthase is substantially lower when compared to 5-fluoro-2′-deoxyuridine (FdU), this effect contributes to the high toxicity of EdU especially at higher EdU concentrations. It results in a lowering of the dTMP, dTDP and dTTP pools and subsequently in the higher incorporation of EdU in DNA.
- EdU induces interstrand crosslinks.
- The use of non-toxic concentrations of EdU (less than 1% cells die using a standard cytotoxicity test) for labelling replicated DNA results in a substantial decrease of the signal when compared to the maximal signal or does not allow any labelling at all. The non-toxic concentration is lower than 0.501 μM, 0.044 μM and 0.47 μM in HeLa, 143B PML BK and HCT116 cells, respectively.

## Materials and Methods

### Cell cultures

Human HeLa cells (cervix, adenocarcinoma; a generous gift from Dr. David Staněk, Institute of Molecular Genetics, Prague), 143B PML BK TK cells (bone, osteosarcoma, contains a herpes simplex virus type 1 thymidine kinase (hsv-1 TK+) plasmid; purchased from ATCC—ATCC CRL-8304) and HCT116 cells (colon, colorectal carcinoma; a generous gift from Doc. Marián Hajdúch, Palacký University, Olomouc) were used. The HeLa cells were cultivated in Dulbecco’s modified Eagle’s medium (DMEM, Gibco), 143B PML BK TK cells (143B) in DMEM supplemented with HAT (0.1 mM hypoxanthine, 400 nM aminopterin and 0.16 mM dT, Sigma Aldrich) and HCT116 cells in McCoy’s medium (Sigma Aldrich) if not stated otherwise. All the media were supplemented with 10% foetal bovine serum (PAA Laboratories), 3.7 g/l of sodium bicarbonate and 50 μg/ml of gentamicin. The cells were cultured in culture flasks or on coverslips (12 mm in diameter) in a Petri dish or in 96 flat bottom well plates (Orange Scientific) at 37°C in a humidified atmosphere containing 5% CO_2_. In the case of the 143B cells, we exchanged the culture medium for a HAT-free medium one week before the experiments. 143B cells containing viral TK were established by the transfection of 143B TK^-^ cells with the vector containing pML-1 plasmid, sequence from the BK virus and hsv-1 TK gene. The transfected 143B PML BK TK cells stably express viral TK [16].

### MTT assay—the survival assay

The MTT assay was performed according to [17] and the instructions of the 3-(4,5-dimethylthiazol-2-yl)-2,5-diphenyltetrazolium bromide (MTT) distributor (Life Sciences). Briefly, the cells were seeded at the density of 5 × 10^3^ cells per well in 96 well plates and incubated at 37°C in a humidified atmosphere containing 5% CO_2_ for 24 hours. The tested nucleosides and inhibitors were added to the culture media and the cells were further incubated for 48 hours. Serial fivefold dilutions of EdU and FdU were used starting at 0.00064 μM and ending at 50 μM. In some cases, we used EdU in combination with 8 nM FdU or 400 nM aminopterin with 0.1 mM hypoxanthine. Then, the culture media were exchanged for nucleoside- and inhibitor-free media and the cells were incubated for an additional 72 hours. The freshly-prepared 1 mM MTT was added and the samples were incubated for 3 hours at 37°C in a humidified atmosphere containing 5% CO_2_. The culture media were removed (except 25 μl) and 50 μl of DMSO was added to each well. The samples were incubated for 10 minutes at 37°C and 300 rpm in the Thermomixer chamber (Eppendorf). Absorbance was measured using a PerkinElmer EnVision Plate Reade (Perkin Elmer) at 540 nm. The measurements were performed for three independent experiments. The graph was constructed using the standard four parameter logistic nonlinear regression (GraphPad Prism 6) according to the following equation: $y=bottom+\frac{top-bottom}{1+10^{\left((logIC50-x)\times hillslope\right)}}$, where *x* is a log of the concentration used; *y* is a measured response; *top* and *bottom* are plateaus in the same units as *y*; *hillslope* is a slope factor. IC50 is a half maximal inhibitory concentration. The data are presented as mean values ± SEM.

### Microscopic analysis of EdU incorporation

The cells were incubated with 0.016; 0.08; 0.4; 2; 10 or 50 μM EdU alone or together with 8 nM FdU for 8 hours. After fixation and permeabilization, the incorporated EdU was visualised by means of a click reaction using Alexa Fluor 488 azide (30 min, room temperature (RT), Life Sciences). The nuclear DNA was stained by DAPI (10 μM, 30 min, RT). The images were obtained by an Olympus IX81 microscope (objective: UPLFLN 10× NA 0.3) equipped with a Hamamatsu ORCA II camera with a resolution of 1344×1024 pixels using Cell∧R acquisition software (Olympus). The data were analysed using CellProfiller image analysis software, Microsoft Excel and the final graphs were made in GraphPad Prism 6 software. The measurements were performed for the three independent experiments. The graph was constructed using the standard four parameter logistic nonlinear regression according to the following equation: $y=bottom+\frac{top-bottom}{1+10^{((logEC50-x)\times hillslope}}$, where *x*, *y*, *top*, *bottom and hillslope* represent the same parameters as mentioned above; EC50 is a half maximal effective concentration. The data are presented as mean values ± SEM. 10,000 cells were evaluated for every cell line.

### Analysis of nucleotide pools by HPLC-MS

The HeLa cells were incubated with 10 μM EdU or 8 nM FdU or without any inhibitor (control) for 2 hours. Next, the cells were harvested and quenched according to the published protocol [18]. Lyophilized cell extracts were dissolved in MeOH/H_2_O (vol. 1:1, 200 μL), centrifuged (10,000 x *g*, 5 min.) and transferred to glass vials. All samples were analysed by HPLC (UHPLC Dionex Ultimate 3000 RS, Thermo Fisher Scientific, MA, USA) using the previously published HILIC method on Luna NH2 (Phenomenex) columns under alkaline separation conditions [19]. For the detection of selected nucleotides, the adopted method using the triple quadrupole mass spectrometer 5500 QTrap (AB Sciex, CA, USA) working in polarity switching multiple reaction monitoring mode was applied [20]. The data were acquired using Analyst 1.6.2 software and processed by MultiQuant 2.1.1. (AB Sciex, CA, USA) and Microsoft Excel. The experiments were conducted in triplicate.

### Doubling time and the relative length of S phase

The cells were seeded in the culture flasks with the grid on the bottom (culture area 25 cm^2^, Greiner Bio-One) and cultivated for 24 hrs. The initial number of cells (0 hr.) was calculated using wide-field microscopy (Olympus IX81 microscope, UPLFLN 10× NA 0.3 objective, Cell∧R acquisition software). 20 pictures were taken for each treatment. The cells were then incubated in the medium containing EdU or EdU and FdU or without any treatment at 37°C in a humidified atmosphere containing 5% CO_2_. At the specific time points, 20 pictures were taken for each sample from the same areas as in the initial time and the number of cells was calculated. The doubling times were calculated using the following formula: $DT=T\times \frac{ln2}{ln(\frac{x_{e}}{x_{b}})}$. The parameter *T* is the incubation time, *x*_*b*_ is the cell number at the beginning of the incubation time, *x*_*e*_ is the number of cells at the end of the incubation time. The experiments were performed in triplicate.

The relative length of the S phase was determined on the basis of the determination of the fraction of EdU-labelled cells after 1-hour labelling with 10 μM EdU as compared to the overall number of the cells. The detection of incorporated EdU and visualization of nuclear DNA was performed as described above. The images were obtained using an Olympus IX81 microscope (UPLFLN 10× NA 0.3). The data were analysed using CellProfiller image analysis software and Microsoft Excel. 10,000 cells were evaluated for every cell line and the experiments were conducted in triplicate.

### Analysis of the cell cycle and BrdU detection

The cells were incubated on glass coverslips in a Petri dish at 37°C in a humidified atmosphere containing 5% CO_2_ with or without 10 μM EdU for 4, 8, 16, 24, 32 and 40 hours. In the case of the analysis of the DNA replication, the cells were incubated with or without 10 μM EdU for the above-mentioned times followed by a 1-hour incubation with 10 μM BrdU. After fixation and permeabilization, the DNA was stained with DAPI (see Section 2.3) or propidium iodide (PI; 40 μg/ml; 30 min). In the case of PI, incubation with 100 μg/ml RNase A for 30 min at 37°C preceded the incubation with PI. Both stains provided similar results. As DAPI did not require treatment with RNAse A, we used DAPI in all of the experiments.

The incorporated BrdU was revealed using copper(I) ions [11] and primary anti-BrdU (Mo-Bu 1, 1:200, 30 min, RT, Exbio) and secondary anti-mouse antibody (Alexa Fluor 488 conjugated, 1:100, 30 min, RT, Jackson Immunoresearch) supplemented with DAPI. Although we used anti-BrdU antibody that should not react with EdU [21,22], we observed a weak signal also in the case when we incubated the cells with EdU exclusively. Therefore, we effectively supressed the EdU-derived signal by 2-azidoethanol and azidomethylphenylsulfide in the double labelling experiments [22]. The images were obtained by an Olympus IX81 microscope (UPLFLN 10× NA 0.3). The data were analysed using CellProfiller image analysis software and Microsoft Excel. 20,000 cells were evaluated in every experiment. The experiments were performed in triplicate.

### Reverse comet assay

HeLa cells were seeded in a 24 well plate (Orange Scientific) in a concentration of 10^5^ cells/ml. The cells were incubated in the culture medium supplemented with 10 μM EdU or 10 μM EdU and 8 nM FdU or 8 nM FdU for 24 and 48 hrs. In the control samples, DMSO was added instead of EdU/FdU. The comet assays were performed according to [23] with the modification of Dr. Dušínska (http://www.cometassayindia.org/Dusinska-protocol.PDF). Briefly, after incubation of the samples with or without the tested nucleosides, the cells were harvested, rinsed and re-suspended in 1× PBS. Then, approximately 1 x 10^4^ cells were re-suspended in 1% low melting point agarose (Sigma Aldrich) and spotted to agarose-coated slides. Agarose-embedded cells were incubated in 25 μM H_2_O_2_ for 5 minutes at 4°C, rinsed in 1× PBS and slowly immersed in the cold lysis solution (2.5 M NaCl, 100 mM Na_2_EDTA, 10 mM Tris base, pH 10) supplemented with 1% Triton X-100 prior to use for 1 hour at 4°C. The samples were then placed in an electrophoresis buffer (300 mM NaOH, 1 mM EDTA, pH>13) for 40 min. Then, a 25 V/300 mA current have been applied for 30 min. followed by neutralization in a neutralization solution (0.4 M Tris, pH 7.5). The DNA was stained with SYBR Gold dye (Invitrogen) for 5 min. The images were obtained using an Olympus IX81 microscope (UPLFLN oil 20× NA 1.3). For each gel, 25 comets were analysed using CometScore (TriTek Corp.) software. The final data evaluation and graph were made using Microsoft Excel software. Each experiment was performed in triplicate.

## Results

### EdU cytotoxicity positively correlates with EdU incorporation efficiency

**Fig 1:**
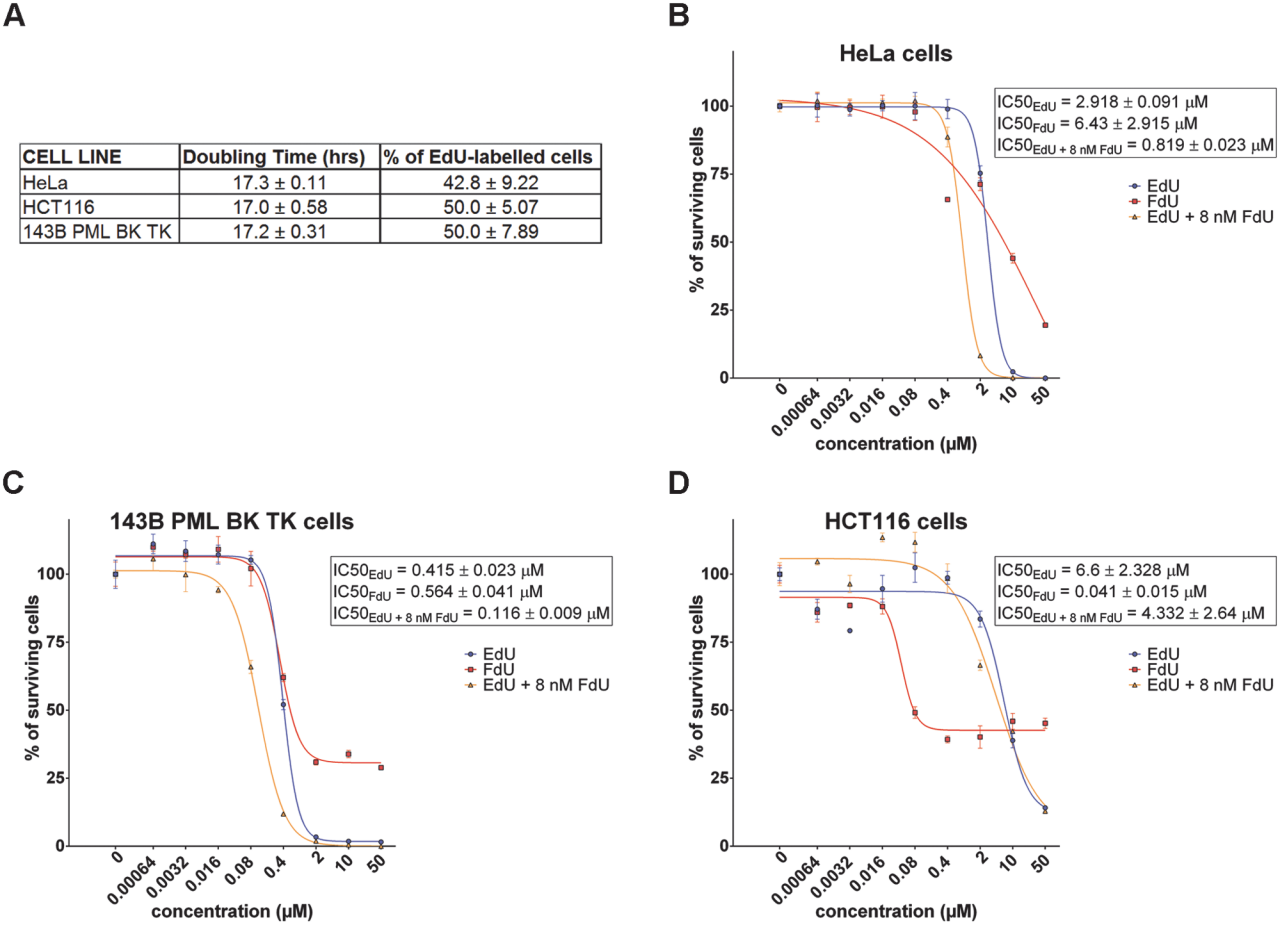
The doubling times and MTT assay results. A. The doubling times and the fraction of EdU-labelled nuclei of HeLa, HCT116 and 143 PML BK TK cells after 1-hour incubation in the culture medium containing 10 μM EdU. The data are shown as the mean ± SEM. B-D. The results of the MTT assay. The impact of EdU, FdU and combination of EdU and 8 nM FdU on HeLa (B), 143B PML BK TK (C) and HCT116 (D) cells are shown. The data represents the mean ± SEM.

We tested the effect of EdU on the cell growth of three human cell lines (HeLa cells, HCT116 cells and 143B PML BK TK cells) using an MTT assay. We have chosen cell lines with similar doubling times and S phase length to exclude the effect of this parameter in the experiments (Fig. 1A). We included the thymidine kinase (TK^-^) negative cell line expressing herpes simplex virus type 1 thymidine kinase (143B PML BK TK; 143B) to evaluate the effect of the viral thymidine kinase on cell viability and EdU incorporation. The doubling time was around 17 hours for all of the cell lines. We found that around 43, 50 and 50% cells in the case of HeLa, HCT116 and 143B cells, respectively incorporated EdU after a 1-hour incubation in an EdU-containing medium. We received similar results if BrdU was used instead of EdU (not shown). As 100% of cells incorporated BrdU in DNA after 18-hour incubation in medium containing 10 μM BrdU, it indicated that all of the cell lines exhibited a similar S phase length.

**Fig 2:**
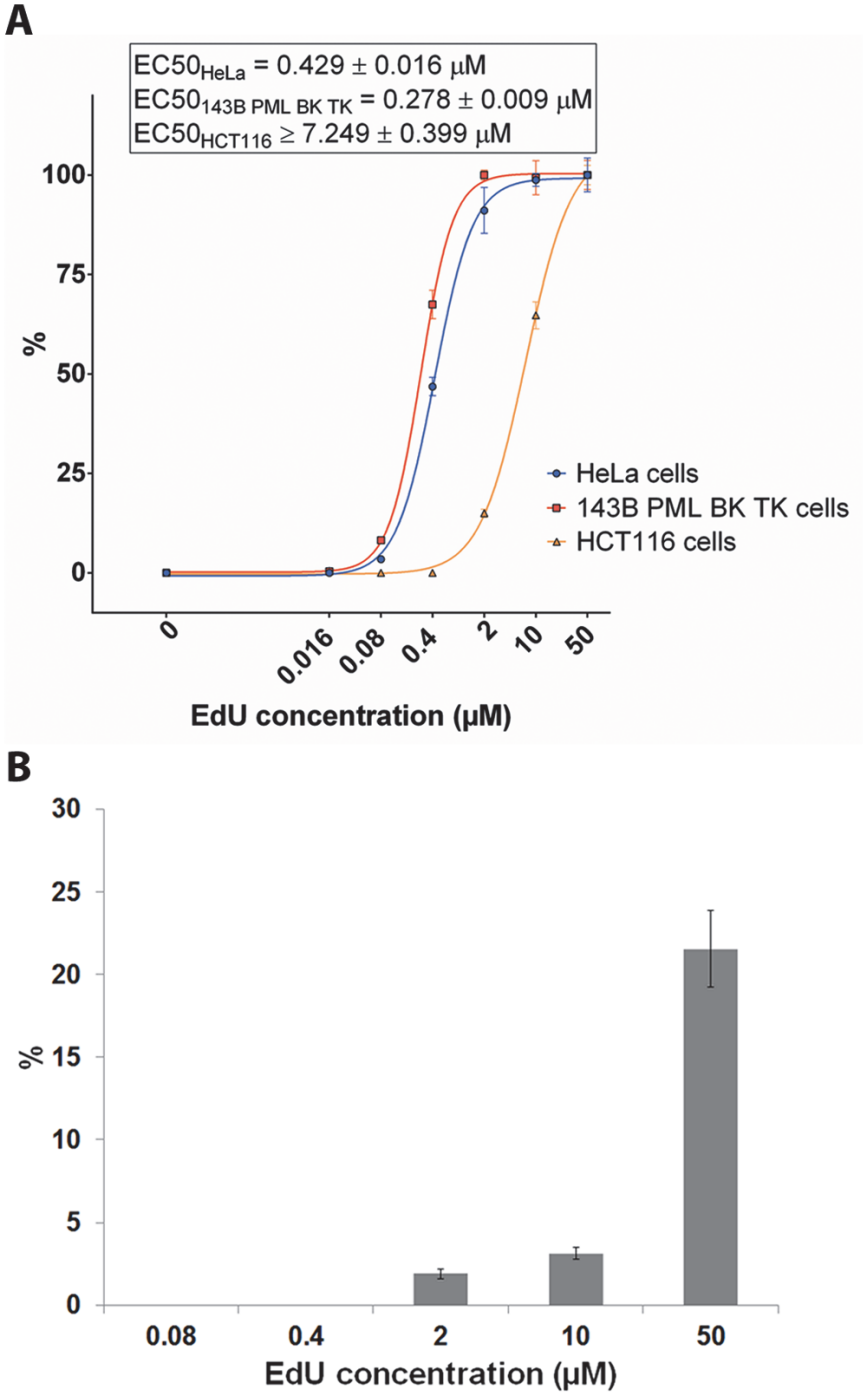
EdU incorporation and effect of dT on EdU incorporation. A. The efficiency of EdU incorporation. The EdU-derived signal in % is plotted against the EdU concentration. The data are normalized to % of the signal of a 50 μM concentration of EdU. The cells were incubated with EdU for 8 hours which was detected using a click reaction with Alexa Fluor 488 azide. EdU-derived signal is equal to the average value of the mean signal in the 5% of the most highly-labelled nuclei. The data are shown as the mean ± SEM. B. The effect of dT on EdU incorporation in DNA of HeLa cells. The ratio between the EdU-derived signal obtained from cells incubated concurrently with EdU and dT and cells incubated with EdU alone is plotted in % against the EdU concentration. The cells were incubated in both cases for two hours in the presence of EdU or EdU and dT and the detection of the incorporated EdU was performed with Alexa Fluor 488 azide. The EdU-derived signal is equal to the average value of the mean signal in thirty percent of the most highly-labelled nuclei. The data are shown as the mean ± SEM.

In order to determine IC50_EdU_, the cells were incubated in culture media containing EdU in a concentration range from 0.00064 μM to 50 μM (fivefold dilutions) for 48 hours. The EdU-containing medium was then removed and the cells were cultivated in an EdU-free media for an additional 72 hours and MTT assay was performed. According to the results IC50_EdU_ was 2.918 ± 0.091; 0.415 ± 0.023; and 6.6 ± 2.328 μM for HeLa, 143B and HCT116 cells, respectively (Fig. 1B-D). As the different sensitivity to EdU can be the result of a different efficiency of EdU incorporation, we determined the EdU concentration that provides half of the maximal EdU signal (EC50_EdU_). The cells were incubated with EdU in the concentration range from 0.016 μM to 50 μM EdU concentration (fivefold dilutions) for 8 hours and the incorporated EdU was visualised with Alexa Fluor 488 azide, cell nuclei by DAPI staining. To determine the degree of the EdU incorporation, we used the mean from the 5% of the most labelled cell nuclei. We used this option to evaluate only such cells that incorporated EdU during a substantial part of the labelling pulse and, simultaneously, had not yet divided in mitosis. It is clear from the graph in Fig. 2A that the particular cell lines differently incorporate EdU in DNA. In HeLa cells, we observed the first detectable signal at the 0.08 μM EdU concentration and the signal significantly increases up to 10 μM. The first measurable signal in 143B cells was measured at 0.08 μM as well; however, the signal grew significantly only up to 2 μM. In HCT116 cells, we observed the first significant signal at a 2 μM EdU concentration; the signal gradually increases up to 50 μM concentration of EdU. EC50_EdU_ derived from the curves were 0.429 ± 0.016 and 0.278 ± 0.009 μM for HeLa and 143B cells, respectively. In HCT116 cells, the value of EC50_EdU_ is apparently above 7.249 ± 0.399 μM. The lower EC50_EdU_ reflects a higher efficacy of EdU incorporation. The fact that cells with higher IC50_EdU_ exhibited higher EC50_EdU_, indicated that one of the factors of the different sensitivity of cells to EdU is a different efficacy of EdU incorporation. It was especially evident in the case of the HCT116 cell line that incorporated EdU at measurable levels at concentrations more than five times higher than HeLa or 143B cells. On the other hand, our measurements also showed that the efficiency of EdU incorporation is not the only factor contributing to the differences in EdU toxicity between various cell lines. The highest incorporation of EdU in the 143B cell line expressing viral TK indicated that the type and/or expression level of TK plays an important role in the toxic effect of EdU on cells.

### The 2′-deoxythymidine metabolism plays a crucial role in EdU toxicity

The efficiency of EdU incorporation should be a function of the dTMP concentration as dTMP is an important competitor at the level of 5-ethynyl-2′-deoxyuridine monophosphate (EdUMP) and 5-ethynyl-2′-deoxyuridine diphosphate phosphorylation and EdUTP incorporation in DNA. dTMP is produced by thymidine kinase from dT and by thymidylate synthase from 2′-deoxyuridine 5′-monophosphate (dUMP; [24]).

### Addition of 2′-deoxythymidine efficiently blocks EdU incorporation

We analysed the effect of elevated levels of dT on EdU incorporation and the effect of thymidylate synthase inhibitors on EdU toxicity and incorporation. We incubated HeLa cells with EdU or with EdU (concentration range from 0.016 to 50 μM; fivefold dilutions; in both cases) and dT (10 μM) for two hours and measured the mean fluorescent signal in the labelled cell nuclei. As significantly more than 30% of cells exhibited a signal in all experiments, 30% of the highest labelled cell nuclei was evaluated and the signal was averaged. Next, we calculated the percentage share (S) of the mean signal in the cells cultivated with both EdU and dT or with EdU exclusively according the following formula: $S=\frac{EdU_{dT}}{EdU}\times 100$, where *EdU*_*dT*_ is the average signal of the mean nuclear signals in the cells cultivated with EdU and dT; *EdU* is the average signal of the mean nuclear signals in the cells cultivated with EdU exclusively. The S value reflected the changes of EdU incorporation in the presence of dT. We found that the presence of dT significantly decreased the rate of EdU incorporation (Fig. 2B). In the case of 2 μM EdU and dT, EdU incorporation was decreased to ca 3% of the signal of the control cells incubated with 2 μM EdU alone. In the case of 50 μM EdU the signal decreased to ca 20% of the signal in control sample. Although we cannot exclude that the decrease of the signal resulted from the different level of the transport of EdU and dT in cell nuclei, we suppose that it is more likely the consequence of the lower ability of cells to phosphorylate EdU to EdUTP or to incorporate EdUTP in DNA.

### The inhibition of thymidylate synthase results in higher incorporation and toxicity of EdU

For the specific analysis of the effect of dTMP on EdU toxicity and incorporation, we inhibited the activity of thymidylate synthase using FdU. FdU is believed to act mainly through the formation of 5-fluoro-2′-deoxyuridine-5′-monophosphate that inhibits thymidylate synthase, an enzyme required for de novo synthesis of pyrimidines [25]. However, its effect is wider as its triphosphate form can be incorporated, although very inefficiently, in newly synthesized DNA and can induce single- and double-stranded DNA breaks [25,26]. The Ki for an FdUMP inhibition of thymidylate synthase is 2 nM [27]. The used FdU concentration was chosen in such a way to be sufficiently below the IC50_FdU_ of all of the cell lines (Fig. 1B-D). The reason for such low FdU concentration was also the fact that FdU could compete with EdU at higher concentrations and cause alternative DNA damages. The measured IC50_EdU+FdU_ in the case of the simultaneous addition of EdU and FdU clearly showed that in all cases IC50_EdU+FdU_ was significantly decreased: 0.819 ± 0.023; 0.116 ± 0.009 and 4.332 ± 2.64 μM for HeLa, 143B and HCT116 cells, respectively (Fig. 1B-D). Interestingly, the IC50_FdU_ was higher than IC50_EdU_ or IC50_EdU + FdU_ in the case of HeLa and 143B cells, but lower in the case of HCT116 cells.

**Fig 3:**
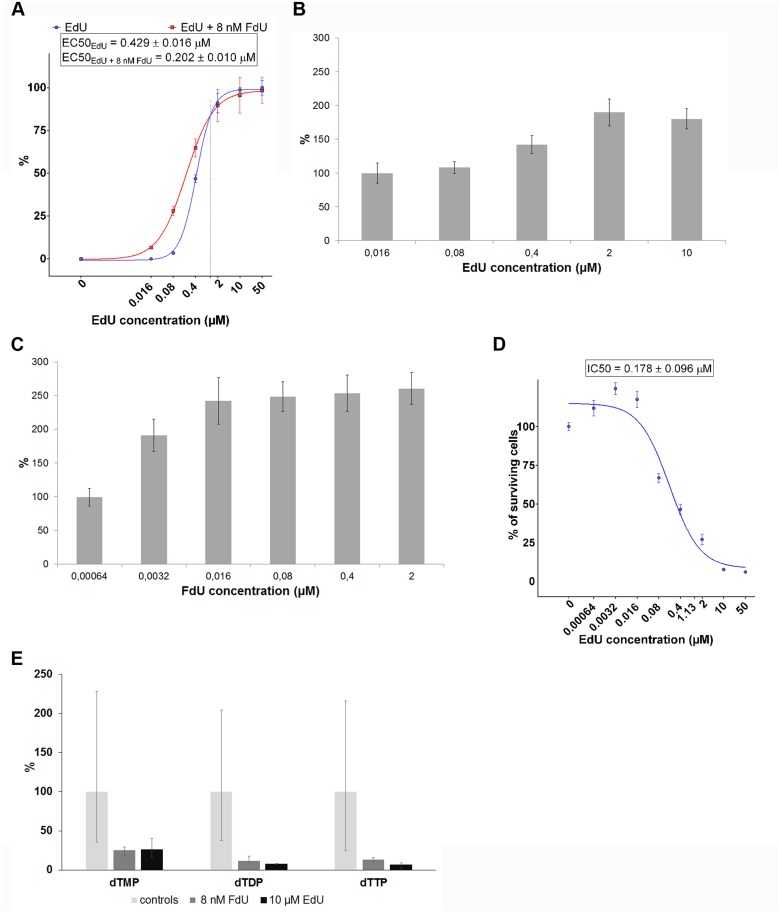
EdU and dT metabolism. A. The effect of FdU on the incorporation efficiency of EdU. The EdU-derived signal in percent is plotted against the EdU concentration. The data are normalized to % of the signal of 50 μM concentration of EdU. HeLa cells were incubated either with EdU or concurrently with EdU and 8 nM FdU for 8 hours and the detection of incorporated EdU was performed using a click reaction with Alexa Fluor 488 azide. The EdU signal is equal to the average value of the mean signal in thirty percent of the most highly-labelled nuclei. The data are shown as the mean ± SEM. B. The effect of EdU on the incorporation of BrdU. The BrdU-derived signal in percent is plotted against the EdU concentration. The data are normalized to % of the BrdU signal of the control non-EdU-treated cells. The HeLa cells were incubated with various concentrations of EdU and 0.4 μM BrdU for two hours. The incorporated BrdU was detected using an anti-BrdU antibody (clone MoBu-1). The BrdU signal is equal to the average value of the mean signal in thirty percent of the most highly-labelled nuclei. The data are shown as the mean ± SEM. C. The effect of FdU on the incorporation of BrdU. The BrdU-derived signal in percent is plotted against the FdU concentration. The data are normalized to % of the BrdU signal of the control non-FdU-treated cells. The HeLa cells were incubated with various concentrations of FdU and 0.4 μM BrdU for two hours. The detection of the incorporated BrdU was performed using an anti-BrdU antibody (MoBu-1). The BrdU signal is equal to the average value of the mean signal in thirty percent of the most highly-labelled nuclei. The data are shown as the mean ± SEM. D. The effect of 400 nM aminopterin on the EdU-mediated toxicity in HeLa cells. The data are shown as the mean ± SEM. The cells were cultured in the culture medium containing 400 nM aminopterin, 0.1 mM hypoxanthine and serial fivefold dilutions of EdU for 48 hours. Further, the medium was exchanged for the inhibitor and the nucleoside-free medium and cells were cultivated for additional 72 hours. Then, MTT assay was performed as described in the Material and Methods. E. The effect of EdU and FdU on the dTMP, dTDP and dTTP pools. The relative changes of the thymidine nucleotides levels in the control HeLa cells and HeLa cells treated with 10 μM EdU or 8 nM FdU is shown. The data are shown as the mean ± maximal and minimal measured value.

We also determined the EC50_EdU+FdU_ (Fig. 3A). We incubated HeLa cells with various concentrations of EdU and 8 nM FdU for two hours, measured the mean nuclear signal of the 30% of the highest labelled cell nuclei, averaged it, analysed the data and calculated EC50_EdU+FdU_. The EC50_EdU+FdU_ was 0.202 ± 0.010 μM while the EC50_EdU_ was 0.429 ± 0.016 μM. The fact that the addition of FdU leading to the approximately 3.6-fold decrease of IC50 was accompanied by a 2.1-fold decrease of EC50 for EdU incorporation clearly documented the direct correlation between the EdU incorporation and its toxicity.

### EdU effectively inhibits thymidylate synthase at micromolar concentrations

EdUMP has been previously shown to be an inhibitor of thymidylate synthase with Ki 0.1 μM as well [28]. Since it is not clear what the concentration of EdUMP in cells is, it is not possible to deduce the degree of the thymidylate synthase inhibition. As 8 nM FdU increases the EdU incorporation only to an ~1.126 μM EdU concentration (see Fig. 3A), it indicates that this Edu concentration inhibits thymidylate synthase as efficiently as the mixture of 8 nM FdU and 1.126 μM EdU. It seems that such a concentration can nearly completely inhibit thymidylate synthase activity. In order to address this issue more specifically, we tested the effect of FdU and EdU on BrdU incorporation (Fig 3B, C). BrdU is widely used as a replication and proliferation marker and is incorporated into DNA very well. In these experiments, we incubated cells with 0.4 μM BrdU in the presence of various concentrations of EdU or FdU for two hours and measured the mean signal of the BrdU in the cell nuclei. We proceeded in the same way as in the analysis of the EdU signal in Fig. 2A. We plotted the BrdU-derived signal of control, non-treated, cells in percent against the EdU/FdU concentration. The BrdU signal was not affected by the presence of 0.016 μM EdU. The first increase of the BrdU signal was observed at an EdU concentration of 0.08 μM (~108% of the signal measured in the control cells). The BrdU signal further increased in the case of 0.4 μM (~142%) and 2 μM EdU concentrations (~190%). When 10 μM EdU was used, the BrdU signal slightly decreased (180%; Fig. 3B). In the case of FdU, the first increase of the BrdU signal was observed after 0.0032 μM FdU (~191%, Fig. 3C). The BrdU signal significantly increased up to a 0.016 μM FdU concentration (~242%) and grew slowly up to a 2 μM FdU concentration (~260%). The decrease of the signal in the case of EdU was apparently a result of the competition of EdU and BrdU at higher concentrations of EdU. This competition can also explain the fact that the FdU addition resulted approximately in up to a 260% increase of the signal while EdU only resulted in a 180% increase. From this point of view, we can conclude that our results showed a similar influence on BrdU incorporation of 0.4–2 μM EdU as 0.0032 and 0.016 μM of FdU.

We have also performed the analysis of the dTMP, dTDP and dTTP pools in the control, non-treated cells, cells treated with 10 μM EdU or 8 nM FdU for two hours (Fig. 3E). Our data clearly showed that the presence of both 10 μM EdU and 8 nM FdU results in the progressive lowering of dTMP, dTDP and dTTP pools (Fig 3E). These data strongly indicate that EdU is an effective inhibitor of thymidylate synthase activity in vivo at micromolar concentrations.

### The inhibition of tetrahydrofolate synthesis increases the EdU toxicity

The increased sensitivity of HeLa cells to EdU in the case of the down-regulation of dT synthesis was further confirmed by the experiment where dT synthesis was inhibited by means of 400 nM aminopterin (Fig. 3D). Aminopterin is an analogue of folic acid that inhibits the activity of the enzyme dihydrofolate reductase [29]. It results in the depletion of tetrahydrofolate which donates one carbon group during the conversion of dUMP to dTTP [30]. As the presence of aminopterin results also in the blockage of purine synthesis, hypoxantine was added to bypass the synthesis of dGTP and dCTP. In the control cells, dT was added together with hypoxantine to bypass the lack of this nucleoside. The presence of aminopterin in HeLa cells led to a considerable increase of EdU cytotoxicity, IC50_EdU+aminopterin_ was 0.178 ± 0.096 μM (IC50_EdU_ was 2.918 ± 0.091 μM). Although the used concentration of aminopterin also decreased the cell growth in the cells cultivated in the culture medium with aminopterin and hypoxanthine without dT to 48.5%, the effect of the addition of EdU was much higher. Importantly, no effect of aminopterin on the doubling time was observed in the cells cultivated in the culture medium supplemented with aminopterin, hypoxanthine and dT.

In summary, our data showed that the EdU toxicity inversely correlated with the activity of the thymidylate synthase. Importantly, our results indicated that, although EdU acts as a relatively weak thymidylate synthase inhibitor, it can substantially contribute to the incorporation of EdU via a decreased rate of dT synthesis at higher EdU concentrations.

### EdU incorporation results in cell cycle deformation

Next, we studied the effect of EdU on the cell cycle. In most of experiments we used HeLa cells although in some cases 143B and HCT116 cell lines were analysed as well. We used HeLa cells as they exhibited a higher sensitivity to EdU than HCT116 cell line, express the human thymidine kinase and are a very common model in many studies. As all mentioned cell lines are adherent cells, we have used image cytometry (see e.g. [31,32]) instead of flow cytometry. It enabled us to work with adherent cells without the need of additional steps.

### EdU increases doubling time of all cell lines tested

**Fig 4:**
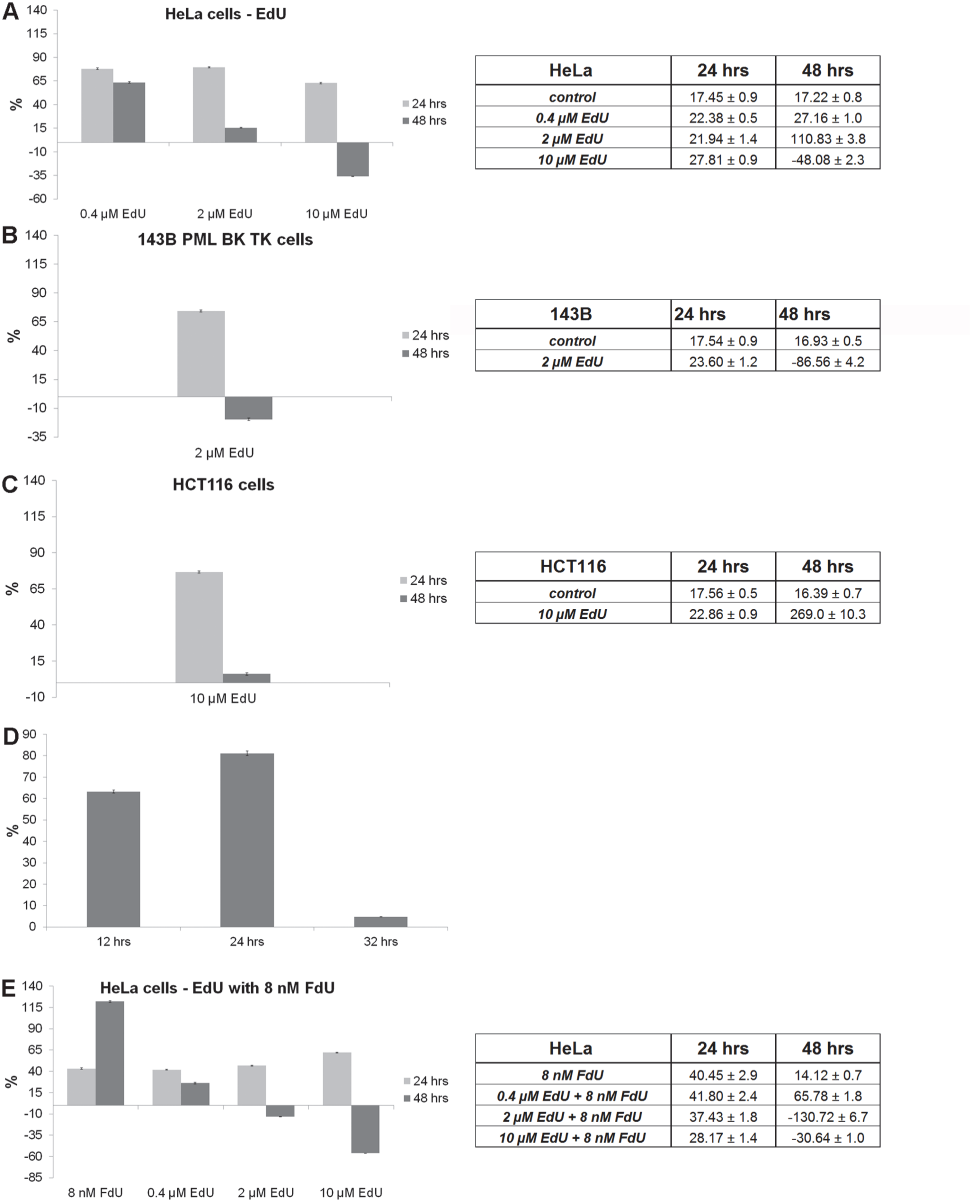
The effect of EdU on the cell growth. A. The effect of 0.4, 2 and 10 μM EdU on the cell growth of HeLa cells between 0th-24th and 24th-48th hour after EdU administration. The value of 1/DT in percent is plotted against the EdU concentration. The data are normalized to % of 1/DT of the control non-treated cells. The table contains the average values of the doubling times. The data are shown as the mean ± SEM.B. The effect of 2 μM EdU on the cell growth of 143B PML BK TK cells between 0th-24th and 24th-48th hour after EdU administration. The value of 1/DT in percent is plotted for the particular time points. The data are normalized to % of 1/DT of the control non-treated cells. The table contains the average values of the doubling times. The data are shown as the mean ± SEM. C. The effect of 10 μM EdU on the cell growth of HCT116 cells between 0th-24th and 24th-48th hour after EdU administration. The value of 1/DT in percent is plotted for the particular time points. The data are normalized to % of 1/DT of the control non-treated cells. The table contains the average values of the doubling times. The data are shown as the mean ± SEM. D. The effect of 10 μM EdU on the cell growth of HeLa cells between 0th-12th, 12th-24th and 24th-36th hour after EdU administration. The value of 1/DT in percent is plotted for the particular time points. The data are normalized to % of 1/DT of the control non-treated cells. The data are shown as the mean ± SEM. E. The effect of 8 nM FdU in the presence of 0.4, 2 and 10 μM EdU on the cell growth of HeLa cells between 0th-24th and 24th-48th hour after EdU administration. The value of 1/DT in percent is plotted against the EdU concentration. The data are normalized to % of 1/DT of the control non-treated cells. The table contains the average values of the doubling times. The data are shown as the mean ± SEM.

First, we analysed the effect of EdU on the cell growth. HeLa cells were cultivated for 24 or 48 hours in medium with or without 0.4, 2 and 10 μM EdU. The doubling times (DT) were determined between the 0^th^ and 24^th^ hrs. and 24^th^ and 48^th^ hrs. (Fig. 4A). We plotted 1/DT ratio as this value directly correlates with cell growth. It is expressed as a percentage fraction of the ratio calculated for the non-treated cells. DTs are shown in the table (Fig. 4A). It is apparent that all of the used EdU concentrations led to the slowdown of cell growth already between the 0^th^ and 24^th^ hour. DT_0–24_ was ca 17.5, 22, 22 and 28 hrs. for the control cells, cells cultivated in 0.4, 2 and 10 μM EdU, respectively. Cell growth was highly deteriorated between the 24^th^ and 48^th^ hours. While the use of 0.4 μM and 2 μM EdU resulted in an increase of DT to ca 27 hrs. and 111 hrs., respectively, the use of 10 μM EdU resulted in a decrease of the number of adherent cells. This observation was completely in agreement with the results of the MTT assay where a 10 μM EdU concentration led to cell death in 100% of cells.

The negative effect of EdU on cell growth was also confirmed in 143B and HCT116 cells. In the case of 143B cells, we observed in accordance with the results of MTT assay a decrease of the number of adherent cells already after the use of 2 μM EdU (Fig. 4B) after 48 hrs. In the case of HCT116 cells evincing much less sensitivity to EdU not even the 10 μM EdU concentration led to a decrease of the number of adherent cells between the 24^th^ and 48^th^ hours (Fig. 4C). On the other hand, DT rose between the 24^th^ and 48^th^ hours to 269 hrs. when compared to ca 17.5 hrs. in the case of the control cells.

To better describe the effect of EdU on cell growth, we performed an analysis of the cell growth in HeLa cells also between 0^th^–12^th^, 12^th^–24^th^ and 24^th^–32t^h^ hrs. (Fig. 4D). In these experiments we incubated cells with a lethal dose of EdU. According to our results, the cell growth in the presence of 10 μM EdU rapidly decreases between the 0^th^ and 12^th^ hours (DT ~28 hrs.), accelerates between 12^th^ and 24^th^ hours (DT ~22 hrs.) and is stopped between the 24^th^ and 32^nd^ hours.

### FdU potentiates the detrimental effect of EdU on cell growth

We tested the effect of the mixture of EdU and 8 nM FdU on cell growth (Fig. 4E). In agreement with our supposition, the negative effect of EdU was amplified both after the 24-hr and 48-hr incubation times. In addition, we observed a decrease in the number of the cells between the 24^th^ and 48^th^ hrs. also in the case of 2 μM EdU. Interestingly, the cell growth between the 0^th^ and 24^th^ hours was inversely proportional to the added EdU concentrations. DT was ca 42, 37 and 28 hrs. for the 0.4, 2 and 10 μM EdU concentrations, respectively. We suppose that it resulted from the lack of dT when dTTP was replaced with EdUTP and this effect partially reversed the negative effect of EdUTP on the cell growth. It is in agreement with the observation that the addition of FdU led to the prolongation of DT to ca 40.5 hrs. Subsequently, the cell growth of FdU-treated cells was sharply accelerated as DT between 24^th^ and 48^th^ hours decreased to ca 14 hrs. Although this effect was surprising, its mechanism was beyond the scope of this study.

### EdU incorporation results in an accumulation of cells in S phase

**Fig 5:**
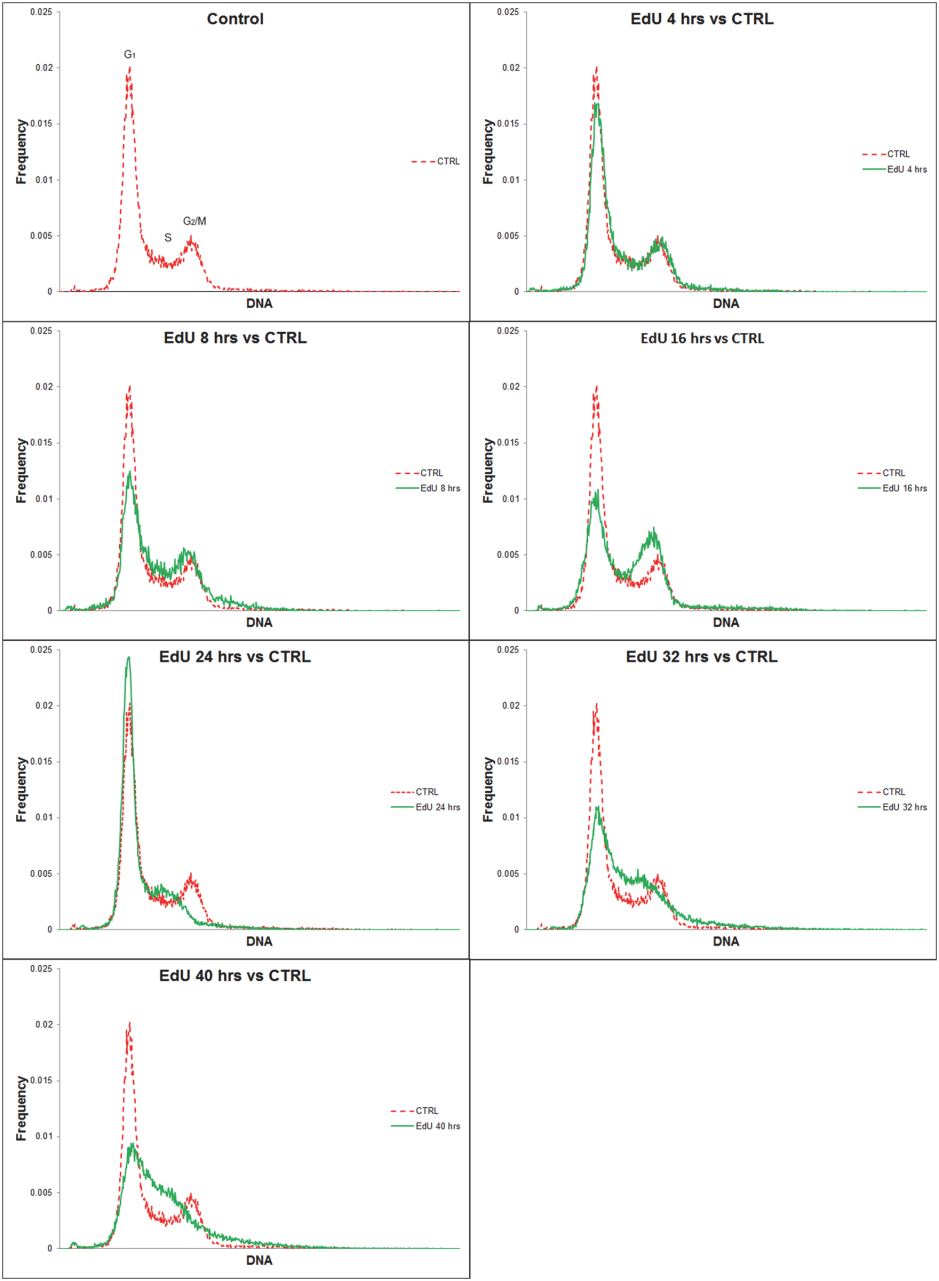
The effect of EdU on the cell cycle of HeLa cells. Cell cycle analysis of HeLa cells incubated with 10 μM EdU for 0, 4, 8, 12, 16, 24, 32, and 40 hours.

The detrimental effect of 10 μM EdU on cell growth was confirmed by a cell cycle analysis (Fig. 5). We observed a slight decrease of the G1 peak height 4 hrs. after the incubation of the cells with EdU. This decrease was further deepened after 8-hr and 16-hr incubations with EdU. On the contrary, we observed the increase of cells in areas corresponding to the S and G2 phase cells. The height of the G1 peak was increased after a 24-hr incubation and the G2 peak almost disappeared. After 32 and 40 hrs., received data indicated substantial accumulation of cells in the S phase. These results together with the results of DT analysis indicated that EdU incorporation initially leads to a slowdown of the progression of the cells through the S and G2 phase, but not to the massive activation of G2 or mitotic checkpoint. The accumulation of cells in the following S phase indicates that the intra S phase checkpoint is probably activated.

### EdU incorporation results in the decrease of DNA synthesis activity

**Fig 6:**
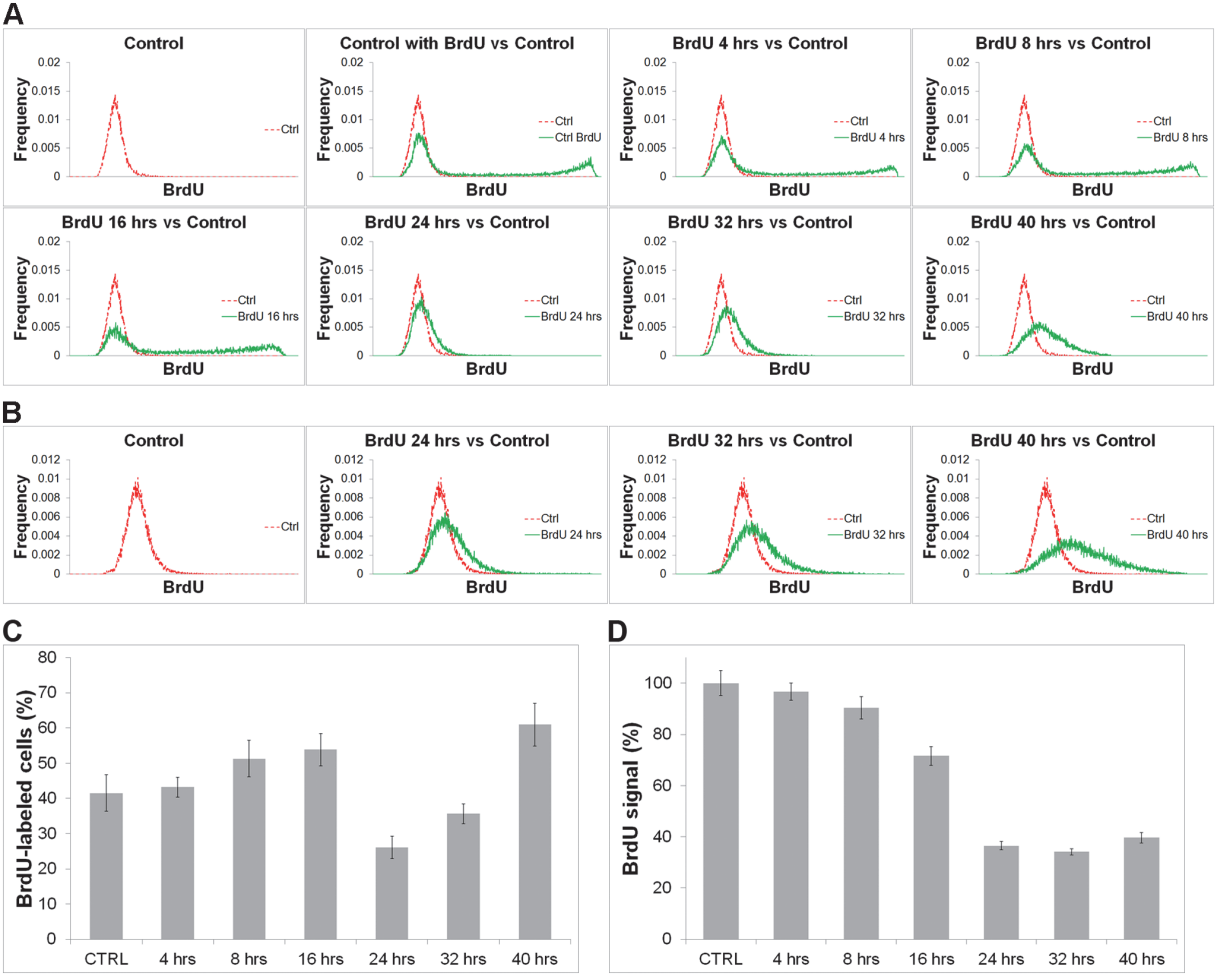
The effect of EdU on DNA replication. A.-B. The effect of EdU on DNA replication. The histograms of the mean signal of BrdU in HeLa cells incubated with 10 μM EdU for 0, 4, 8, 12, 16, 24, 32, and 40 hours followed by a 1-hour incubation with 10 μM BrdU are shown. The acquisition time was 300 ms for the histograms in A and 490 ms for the histograms in B. C. The percentage of the replicated cells deduced from A. and B. 100% corresponds to the total number of the cells. The data are shown as the mean ± SEM.D. The averaged mean nuclear BrdU signal of replicating cells incubated with 10 μM EdU for 0, 4, 8, 12, 16, 24, 32, and 40 hours. The data are normalized to % of BrdU-signal of the control non-EdU-treated cells. The data are shown as the mean ± SEM.

In the next experiments, we studied DNA synthetic activity in HeLa cells. In this case, the cells were first incubated with 10 μM EdU for 0, 4, 8, 16, 24, 32 or 40 hrs. and then with 10 μM BrdU for 1 hour (Fig. 6). In Fig. 6A, a histogram of the mean intensities of the signals in the cell nuclei after the BrdU detection in cells incubated with EdU and in the control non-labelled cells is shown. The acquisition time was 300 ms in all cases. This acquisition time was chosen in such a way that the dynamic range allowed for the maximal resolution of the negative and positive nuclei in the cells incubated in EdU for 0–16 hrs. As after the 24-hr incubation the BrdU signal substantially decreased and the decrease was observed also in cells after 32- and 40-hour incubation times, we used the experimentally optimised acquisition time of 490 ms for these experiments as well (Fig. 6B). To identify the number of labelled nuclei, we subtracted the frequency of every mean value in the treated and control cells and then we summarized all the frequencies that evinced a positive value. We performed this evaluation for all of the incubation times. The obtained data (Fig. 6C) indicated that the number of replicating cells gradually increases from a 4- to a 16-hour incubation in EdU. It increases from ca 42% of replicating cells in the control sample to ca 54% of replicating cells after a 16-hour incubation in EdU. By contrast, after 24 hours, we observed a strong decrease of replicating cells. The fraction of replicating cells decreased to ca 25%, probably as a consequence of cell division which occurs between the 16^th^ and 24^th^ hrs. and the subsequent accumulation of cells in the G1 phase. Next, we observed the gradual increase of the replicating cells to ca 35% and 60% of cells with replicated DNA after 32- and 40-hr. incubations, respectively. These results confirmed our data from the analysis of the DNA content.

Next, we evaluated the mean replication signal per nucleus of replicating cells (Fig. 6D). As a border value, we used the value corresponding to 99% of the least labelled cells in the control non-labelled sample. For the analysis of the mean signal intensity we used the acquisition time 90 ms. This acquisition time did not result in the saturation of the signal in any of the samples. For the identification of replicating cells, we used two optimised times: 300 ms for cells incubated with EdU for 0, 4, 8 and 16 hrs. and 490 ms for cells incubated with EdU for 24, 32 and 40 hrs. In practise, it meant that two acquisition times were used for all of the evaluated cells. The selection of replicating cells was conducted on the basis of the longer time. It is evident that the mean synthetic activity progressively decreased and the highest decrease of the signal was observed after a 32-hr. incubation when it reached 34% of the original value. Although subsequently the synthetic activity slightly increased, it was still below the 40% of the original value. These results indicated that EdU incorporation led to a decrease of the average replication activity.

### Incorporation of EdU results in changes of the DNA

**Fig 7:**
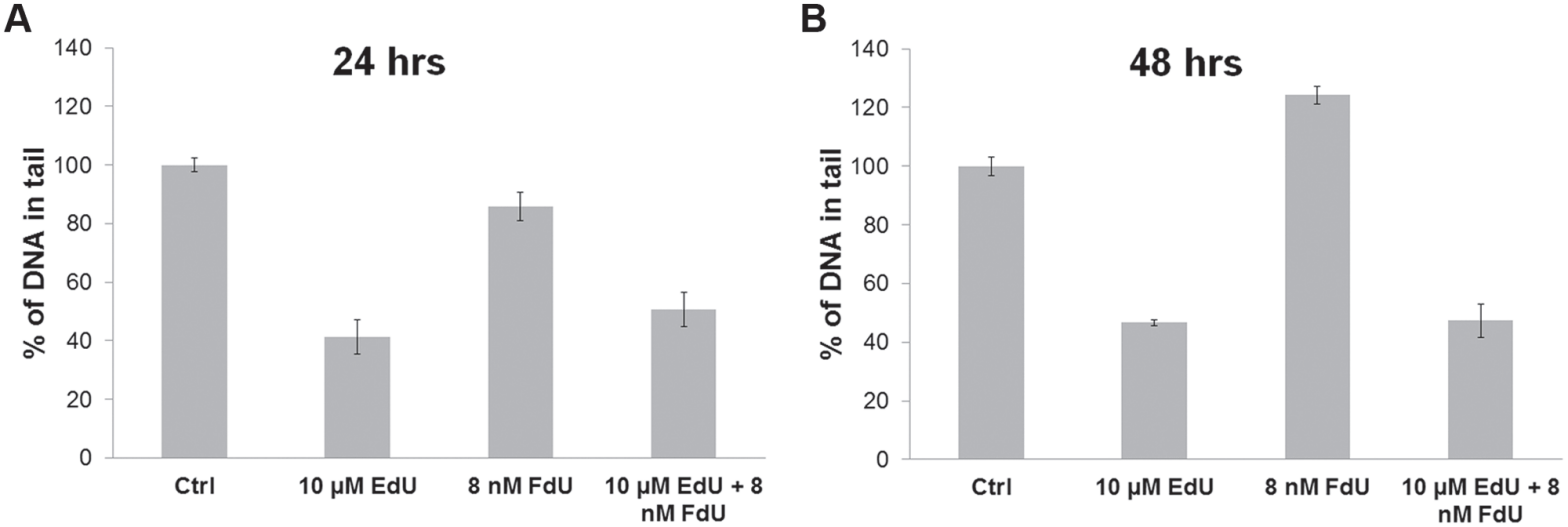
Reverse comet assay analysis of EdU and FdU treated cells. A.-B. Graphs illustrating the percentage of DNA in tails. For the data analysis, the reverse comet assay of cells incubated with 10 μM EdU, 10 μM EdU and 8 nM FdU together and 8 nM FdU for 24 (A) and 48 (B) hrs. was used. The data are normalized to % of tail in the DNA of the control H2O2-treated samples. The data are shown as the mean ± SEM.

We tested the possibility that the presence of EdU in DNA can affect the integrity of cellular DNA. We incubated HeLa cells with or without 10 μM EdU or 8 nM FdU or concurrently 10 μM EdU and 8 nM FdU for 24 or 48 hrs. The cell integrity was analysed using reversed comet assays. This protocol is based on the partial fragmentation of DNA in the presence of hydrogen peroxide and is commonly used for the testing of the DNA interstrand crosslinks (ICL, see e.g. [33]). According to our results, EdU-treated cells exhibited an approximately 2-fold decrease of DNA in a tail comparing to the cells cultivated without EdU after 24-hr incubation and a 2.5-fold decrease after a 48-hr incubation (Fig. 7A, B). A similar amount of DNA was found in the tails of the samples prepared from cells co-treated with FdU (Fig. 7A, B). Our data also showed that DNA in cells treated with FdU in the absence of EdU was slightly more degraded than untreated samples in the case of the 48-hr treatment. It apparently reflected the negative impact of FdU on DNA integrity. On the other hand, the decrease of the amount of DNA in the comet tail in EdU, or EdU plus FdU treated cells may reflect the formation of DNA lesions such as interstrand DNA crosslinks which prevents the unwinding of the DNA under alkali conditions and leads to a decrease of DNA mobility during gel electrophoresis.

The results obtained also indicated that the mechanism of EdU cytotoxicity is strongly connected with the process of DNA replication. We suggest that the cells are able to proceed through the first S phase when they incorporated the supplied EdU in DNA. Then, the incorporated EdU probably induces the formation of DNA adducts which are according to our results from comet assay ICLs. Interstrand covalent bounds are known to be very toxic for cells due to the disruption of DNA replication and RNA transcription processes followed by cell death [34] as most ICLs cannot be repaired by the DNA repairing systems. This is probably one of the reasons why cells treated with EdU are not able to proceed through the second S phase, accumulated in it and gradually die.

### The use of low, non-toxic EdU concentrations results in the progressive decrease of the replication signal

**Fig 8:**
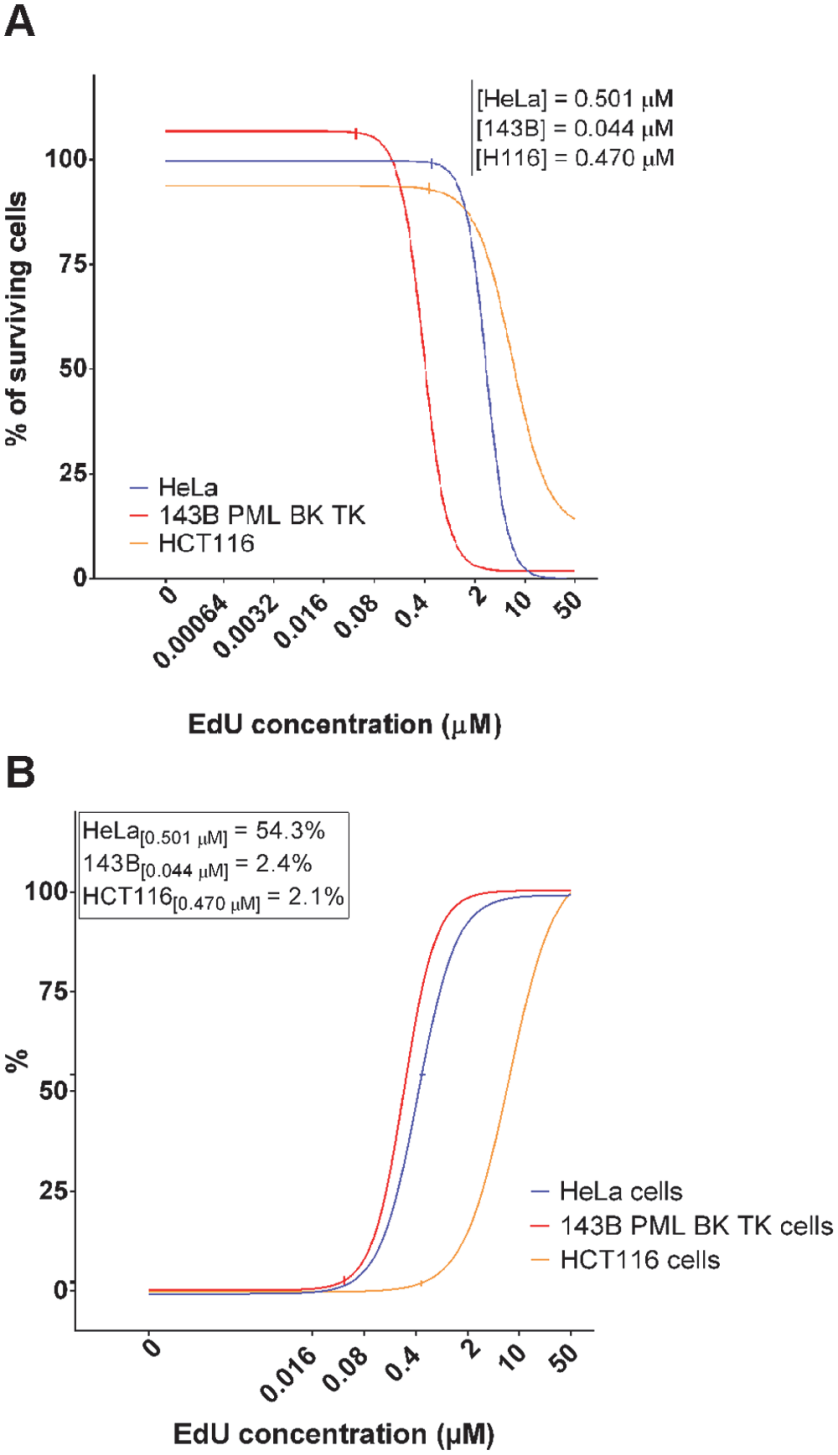
The EdU toxicity and the EdU-derived signal intensity. A. The dependence of EdU toxicity on the EdU concentration for HeLa, 143B PML BK TK and HCT116 cell lines and the border values defining the concentration that provides more than 99% of living cells with respect to the control, non-treated cells. B. The EdU-derived signal intensity dependence on the EdU concentration for HeLa, 143B PML BK TK and HCT116 cells and values of the signal provided by border concentrations from Fig. 8A.

We further assessed the EdU toxicity with respect to its use as a replication marker. Apparently, the ratio between the detectability of any replication marker and its toxicity should be as high as possible. The graph in the Fig. 8A shows the EdU concentrations providing 99% of living cells with respect to the control cells. They are around 0.501 μM; 0.044 μM and 0.47 μM for HeLa, 143B and HCT116 cells, respectively. If these concentrations are used for the detection of replication activity, the obtained signal is 54.3% of the maximal signal in the case of HeLa cells, however only 2.4% and 2.1% for 143B and HCT116 cells, respectively (Fig. 8B).

## Discussion

In the study presented, we dealt with the impact of EdU on cell metabolism. First, we addressed the possibility that there is a direct correlation between the EdU toxicity and the incorporation efficiency. The IC50_EdU_ deduced from the MTT assay were 2.918 ± 0.091; 0.415 ± 0.023; and 6.6 ± 2.328 μM for HeLa, 143B and HCT116 cells, respectively. The EC50_EdU_ of the signal reflecting the EdU incorporation effectiveness was 0.429 ± 0.016 and 0.278 ± 0.009 μM for HeLa and 143B cells, respectively and higher than 7.249 ± 0.399 μM for HCT116 cells. As a lower EC50 reflects the higher efficacy of EdU incorporation and there is an evident relationship between IC50 and EC50 we suggest that the different efficacy of EdU incorporation of cells is the crucial factor that influences EdU toxicity. The highest incorporation efficiency of EdU in 143B cells line expressing viral TK indicated that the type and/or expression level of TK plays an important role in the case of the toxic effect of EdU. It is in agreement with the previous findings showing that EdU inhibits cell proliferation more efficiently in cells expressing viral thymidine kinase [3].

Our data also showed the relationship between dT metabolism and EdU incorporation. We observed a highly negative correlation between dT concentration and EdU incorporation and a negative correlation between thymidylate synthase activity and EdU incorporation. In this respect, already the addition of 8 nM FdU, an inhibitor of thymidylate synthase, resulted in the lowering of the IC50 in all of the cell lines tested. The IC50 decreased to 0.819 ± 0.023; 0.116 ± 0.009 and 4.332 ± 2.64 μM for HeLa, 143B and HCT116 cells, respectively. The simultaneous analysis of the EdU signal in HeLa cells confirmed that the higher toxicity of EdU was accompanied by a higher efficiency of EdU incorporation. Our data also confirmed the previously suggested role of EdU as an inhibitor of thymidylate synthase [5]. In this respect, we have shown that the in vivo effect of EdU on thymidylate synthase activity is much lower than the effect of FdU. We found that 2 μM EdU enhanced the incorporation of BrdU approximately 1.9 times when compared to the control, non-EdU-treated, cells. Such an effect had already been observed in the case of 0.0032 μM FdU concentration. As we have to take into account the competition of EdU and BrdU at the level of phosphorylation and incorporation, it is probably more appropriate to interpret our data as evidence that 0.4–2 μM EdU inhibits thymidylate synthase in vivo in a similar way that 0.0032–0.016 μM FdU does. It is in agreement with our observation that the simultaneous addition of EdU and FdU to cells led to the increased incorporation of EdU in DNA up to ~1.126 μM EdU. Our experiments indicated that the EdU concentration above 1.126 μM results in the nearly complete inhibition of thymidylate synthase activity in vivo in the case of HeLa cells. The analysis of dTMP, dTDP and dTTP pools clearly showed that the presence of 10 μM EdU results in the progressive lowering all of these nucleotides. As dT and its nucleotides represent very strong competitors of EdU, the inhibition of thymidylate synthase apparently increases EdU toxicity as it facilitates the incorporation of EdU in DNA. The inhibitory effect of EdU on thymidylate synthase can also result in imbalances of other DNA precursors. In this respect, it was previously shown that the depletion of dTMP and subsequently dTTP induces perturbations in the levels of the other deoxynucleotides (dATP, dGTP and dCTP) resulting in a disruption of the DNA synthesis and repair [35]. Our results have also shown that EdU incorporation leads to the slowdown and abnormalities of the cell cycle including the slowdown of the S phase accompanied by a decrease in the DNA synthetic activity. We observed the slowdown of DNA replication after a 4-hr. incubation of cells with EdU, however, no decrease was observed after a 2-hr. pulse. It is in agreement with [15] who did not observe a decrease in the DNA synthetic activity in HeLa cells, after 20-min. and 2-hr. incubations of the cells with EdU. Irrespective of the lowering of the DNA synthetic activity, we observed the accumulation of cells in the next S phase. This is in agreement with the data of [13] who observed that the most affected was the progression of cells through the S phase subsequent to that at which they had incorporated EdU. Importantly, our data indicates that EdU causes interstrand crosslinks. Such an irreparable DNA lesion can result in the activation of the intra S phase checkpoint followed by cell death. The activation of the intra S phase checkpoint is also in agreement with results of [36]. They showed that EdU activated the intra S phase checkpoint in yeast cells. Interestingly, this checkpoint was activated not earlier than in the second S phase after the administration of toxic doses of EdU. [12] showed that EdU treatment results in DNA breaks. However, the finding indicating that the ICLs are formed after EdU treatment are not in violation of DNA breaks formation as ICLs can result in apoptotic changes accompanied by double- and single-stranded DNA breaks. Although [13] observed an accumulation of cells in the G2 phase and suggested that the G2 checkpoint is activated, they used a 1-hour labelling pulse with EdU and not the permanent cultivation of cells in an EdU-containing medium. In addition, they used a different cell line than in the study presented here.

Our data have clearly shown that the common EdU concentrations used for the detection of replicated chromatin represent highly toxic doses. On the other hand, the decrease of the concentrations to non-toxic ones results in a substantial decrease of the signal.

In summary, our data showed that EdU can be a very promising drug the effect of which can be increased by thymidylate synthase inhibitors. However, its use as a replication marker is limited to studies using short pulses.
